# A top-down manner-based DCNN architecture for semantic image segmentation

**DOI:** 10.1371/journal.pone.0174508

**Published:** 2017-03-24

**Authors:** Kai Qiao, Jian Chen, Linyuan Wang, Lei Zeng, Bin Yan

**Affiliations:** National Digital Switching System Engineering and Technological Research Centre, Zhengzhou, China; Soochow University, CHINA

## Abstract

Given their powerful feature representation for recognition, deep convolutional neural networks (DCNNs) have been driving rapid advances in high-level computer vision tasks. However, their performance in semantic image segmentation is still not satisfactory. Based on the analysis of visual mechanism, we conclude that DCNNs in a bottom-up manner are not enough, because semantic image segmentation task requires not only recognition but also visual attention capability. In the study, superpixels containing visual attention information are introduced in a top-down manner, and an extensible architecture is proposed to improve the segmentation results of current DCNN-based methods. We employ the current state-of-the-art fully convolutional network (FCN) and FCN with conditional random field (DeepLab-CRF) as baselines to validate our architecture. Experimental results of the PASCAL VOC segmentation task qualitatively show that coarse edges and error segmentation results are well improved. We also quantitatively obtain about 2%-3% intersection over union (IOU) accuracy improvement on the PASCAL VOC 2011 and 2012 test sets.

## 1 Introduction

Semantic image segmentation is one of the central and important computer vision tasks. Compared with image classification aiming at labeling at the image level, semantic image segmentation needs to assign a semantic label at each pixel. Classifying region proposals and refining labels to obtain final segmentation is a common technique. Carreira et al. [1] used constrained parametric min-cuts [2] to generate 150 region proposals per image and then predicted each region with the use of variants of scale-invariant feature transform and local binary pattern. Jimei et al. [3] presented a scalable scene parsing algorithm based on image retrieval and superpixel matching, and obtained good performance. Tighe et al. [4] combined region-level features with per-exemplar sliding window detectors for interpreting a scene. Despite being the focus of considerable attention, such a task remains challenging.

The combination of deep convolutional neural networks (DCNNs) and simple classifiers has led to a series of breakthroughs in image classification task [5–13]. Recently, He et al. [14] proposed the deep residual network with a depth that reached 152 layers and achieved an accuracy of 96.43% on the ImageNet ILSVRC 2015 classification task. Success in the classification task benefits mainly from the powerful hierarchical feature representation of DCNNs. Encouraged by the success, some new applications [15, 16] of deep learning begin to appear. In addition, researchers apply the excellent recognition capability to extract high-level semantic features for structured prediction problems, such as detection [17–20] and semantic segmentation [21–23]. Currently, DCNN-based methods are overwhelmingly considered the state of the art in various computer vision tasks.

According to the results of the PASCAL Visual Object Class (VOC) segmentation benchmark [24, 25], the current best performing methods all use DCNNs. Farabet et al. [26] selected a DCNN model as a multi-scale per-pixel classifier, and Girshick et al. [17] employed a DCNN model to classify multi-scale region proposals generated by the selective search method [27]. Bharath et al. [28] proposed Simultaneous Detection and Segmentation (SDS), and used category-specific, top-down figure-ground predictions to refine bottom-up proposals. By contrast, Long et al. [21] trained an end-to-end, pixel-to-pixel fully convolutional network (FCN) based on DCNNs, which driven recent further breakthroughs in semantic segmentation. The FCN converts an existing DCNN classification model for semantic segmentation by employing deconvolution to unsample the high-level features obtained by hierarchically feed-forwarding an input image. On one hand, this work highlights a simple interpolation filter that is employed for deconvolution and needs not be fixed but could be learned. On the other hand, this work introduces a skippable architecture that combines semantic information and appearance information from deep and shallow layers, respectively, to produce more accurate and detailed segmentations. As a result, an impressive 20% mean intersection over union (IOU) improvement to 62.2% on the PASCAL VOC 2012 segmentation test set is achieved. Then, Chen et al. [29] refined label maps based on FCN with fully connected conditional random field (CRF) to improve segmentation accuracy [30]. Xie et al. [31] employed Convolutional Pseudoprior (ConvPP) for structured labeling, and obtained better performance than FCN. Noh et al. [22] added a symmetry deconvolution network after a VGG [8] convolutional model proposed by “VGG” team to improve segmentation accuracy further.

**Fig 1:**
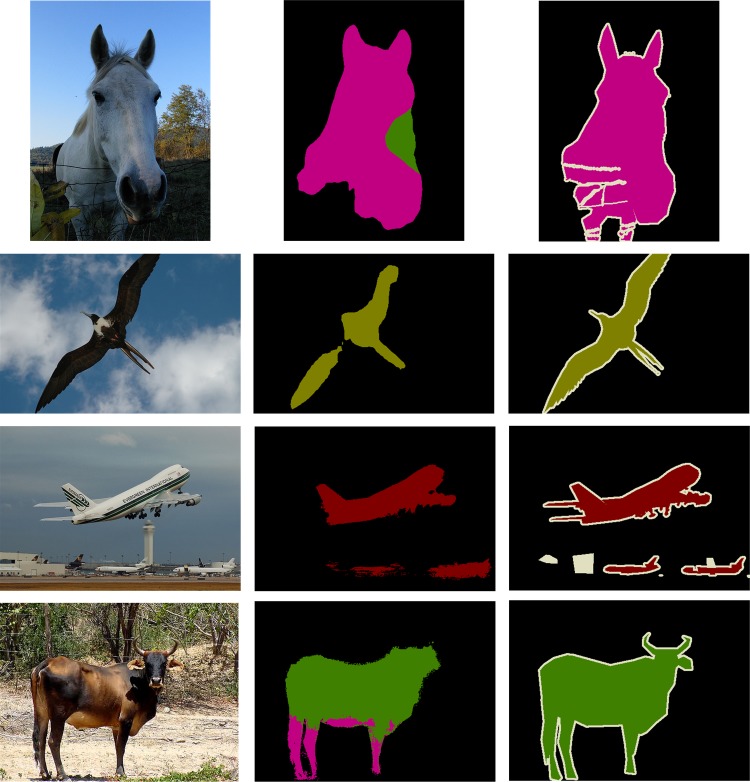
Coarse semantic segmentation results of the PASCAL VOC dataset based on the FCN and DeepLab-CRF model. **Different colors represent different classes.** (a) Input image (b) Segmentation results from FCN (first two rows) and DeepLab-CRF (last two rows) (c) Ground truth.

However, the segmentation results of DCNN-based methods are still coarse and limited, as shown in Fig 1, even if some improvement is achieved owing to the skip architecture [21] and CRF [29, 30].

According to current progress on DCNNs in computer vision tasks, the accuracy of the image classification task is much higher than that of the semantic image segmentation task. Why are DCNNs more successful in classification than in segmentation?

We firstly analyze the above question from the perspective of computer vision., Semantic image segmentation task is more difficult because the classification task refers to as image-level labeling that needs only recognition capability to identify global information, whereas the segmentation task refers to as pixel-level labeling that depends on not only recognition capability but also visual attention capability to consider local detailed information.

On the other hand, progress on the human visual mechanism significantly inspires us. Human can effortlessly process various difficult vision tasks with more than 30 separate visual areas in the cortex [32]. Visual cortical areas appear to be organized mainly into two hierarchically arranged and functionally specialized processing pathways, namely, a ventral stream and a dorsal stream [33, 34]. The ventral cortical area is mainly responsible for recognition, whereas the dorsal cortical area is mainly responsible for spatial location [35–37]. It has proved that the features of high layers in DCNNs are highly predictive of neural responses in the high-level V4 and IT cortices of the ventral cortical area [38, 39]. Given an input image, DCNNs extract hierarchical features in a bottom-up manner and higher features possess more powerful recognition capability. The means of hierarchical DCNNs is similar to the mechanism of the ventral cortical area where hierarchical visual information flow from low-level V1 visual cortical area to high-level IT visual cortical area in a bottom-up manner [32, 40]. These discoveries could powerfully explain why DCNNs can achieve significant success in the image recognition task. Moreover, biased competition theory [41–44] in cognitive science explains that the human visual cortex is enhanced by top-down stimuli to generate visual attention that mainly pays attention to local detailed structure information, such as edges and contours. Given an input image, human firstly globally recognizes it in a bottom-up manner then generates visual attention in a top-down manner and performs detailed identification [45]. So, visual attention plays an important role in processing dense predicting for human visual cortex [46]. However, DCNNs in a bottom-up manner similar to the ventral stream weaken local detailed information while extracting hierarchical abstract features and lack visual attention capability that is essential in segmentation task. Consequently, current methods are faced with coarse segmentation results and DCNN-based methods in a bottom-up manner are not enough for semantic image segmentation.

In view of the preceding biological analysis, we think about producing visual attention in a top-down manner to improve the segmentation accuracy of DCNN-based methods in a bottom-up manner. The combination of visual attention and recognition capability presents complementary of multi-level features, besides, the combination of bottom-up manner and top-down manner creates complementary of pattern.

In contrast to DCNNs, superpixel methods perform clustering segmentation and are good at grasping local detailed structure that consists of similar visual attention information. Considering the recognition and visual attention mechanism, we propose to employ DCNN-based methods in a bottom-up manner to obtain semantic segmentation results, and produce superpixels containing local detailed information in a top-down manner to refine them. Owing to the complementarity of the bottom-up and top-down manners, our proposed architecture is extensible and suitable for other methods that can extract local detailed information or other DCNN-based semantic segmentation model.

Our main contributions are as follows: 1) We find a way based on visual mechanism to explain why DCNNs in a bottom-up manner are not enough for the semantic image segmentation task. 2) We propose an architecture that introduces semantic labels from DCNN-based methods in a bottom-up manner to help produce better superpixels that are conversely used to improve semantic labels in a top-down manner. 3) We achieve a better segmentation performance (3.1% and 1.5% improvement compared with the FCN and DeepLab-CRF) on the PASCAL VOC 2012 test set.

In Section 2, we introduce our top-down architecture. In Section 3, we qualitatively and quantitatively evaluate our performance on the PASCAL VOC segmentation task and then present the discussion. Finally, we summarize our work and suggest future work in Section 4.

## 2 Proposed method

To our knowledge, DCNNs have proved their recognition capability in semantic segmentation task. Cognitive science explains that equally important visual attention is typically dominated by “goals” in a top-down manner. Simulating the visual attention mechanism, we propose a novel architecture that introduces local detailed structure information in a top-down manner based on semantic labels from DCNNs in a bottom-up manner to improve semantic segmentation. We choose the current state-of-the-art FCN [21] and the DeepLab-CRF [29] as baselines, and employ the GS [47] superpixel method with good boundary adherence to further improve segmentation performance of the baselines.

We next explain our architecture. Section 2.2 describes the baselines. In section 2.3, we explain how to produce better superpixels with the help of global semantic information from DCNN-based methods. Overall architecture is overviewed in section 2.4.

### 2.1 Architecture

The proposed architecture is mainly composed of superpixels and the semantic labels based on the DCNN-based methods. Given a single image with the size of *w* × *h*, firstly, we employ the current DCNN-based model to perform coarse prediction at each pixel. The final output is *c* probability maps, where *c* refers to 21 channel dimensions (PASCAL VOC includes 20 object categories and a background). Each probability map has the same size as input image and each value indicates the probability of corresponding pixel belonging to one of the predefined classes. Then, we employ the GS superpixel method to generate superpixels as visual attention. The GS method employs the difference in the colors of neighbor pixels as a measure of the dissimilarity to perform pixel clustering. This method mainly focuses on local structure information and adheres well to object boundaries. We combine semantic labels and local structure information to improve segmentation accuracy. We regard that the pixels from one superpixel belong to the same class, and label each pixel with the average semantic labels of all pixels from the superpixel that contains it, equally labeling each superpixel with the average of the semantic labels of its inner pixels by Eq (1), where *vec*_*i*_ denotes the label vector of pixel *i*, and |*s*| denotes the number of pixels in superpixel *s*.

$$vec_{s}=\frac{1}{\left|s\right|}\sum_{i\in s}vec_{i}.$$ (1)

We employ local detailed structure information from superpixels to refine semantic labels. In a certain pixel label, namely, 21-dimension vector, if the value corresponding to the right class index is not the largest in 21 values, then the segmentation is evaluated as an incorrect result. Accordingly, we can divide segmentation error into two cases. One case is that the value corresponding to the right class index is not the largest but larger. Our architecture could correct it by performing the average because the probability that most neighbor pixels are labeled as the same wrong class is relatively low. The other case is that the value corresponding to the right class index is smaller. Our architecture can still correct it by performing the average if most of the neighbor pixels belonging to the same superpixel are rightly labeled. Introducing superpixels that contain visual attention can help refine coarse semantic segmentation results.

Although the above architecture indeed improves segmentation performance of DCNN-based methods, the combination is simple and not compact. We hold that more compact combination can produce more improvement. When generating superpixels, GS method has nothing to do with semantic labels containing global semantic information which is yet valuable. More importantly, considering human visual mechanism, visual attention is generated in a top-down manner. So, we consider introducing semantic labels from DCNN-based methods to generate better superpixels, and better superpixels help improve the semantic labels. They are interacted and help each other, then develop better semantic segmentation results.

GS method performs an agglomerative clustering of pixels as nodes on a graph. In the graph, each node denotes each pixel, and edges connect neighbor pixels and are measured by Euclidean distance of their colors, i.e.,

$$w\left(\left(v_{i},v_{j}\right)\right)=\sqrt{\sum_{c=r,g,b}{\left(p_{i}\left(c\right)-p_{j}\left(\text{c}\right)\right)}^{2}}$$ (2)

Where *p*_*i*_(*c*) denotes the value of pixel *i* in terms of color channel *c*. For GS method, the final superpixel segmentation results only depend on the edges. The appearance that neighbor pixels have big difference but indeed belong to the same object is very common. Inappropriate measure leads to defective superpixels and reduces the improvement for semantic labels. So, We modify the edge measurement by adding difference of semantic labels, i.e.,

$$w\left(\left(v_{i},v_{j}\right)\right)=\sqrt{\sum_{c=r,g,b}{\left(p_{i}\left(c\right)-p_{j}\left(c\right)\right)}^{2}}+t\cdot \mu \left(l_{i},l_{j}\right)$$ (3)

where *l*_*i*_ denotes the label of pixel *i* and *t* denotes constant parameter; *μ*(*l*_*i*_,*l*_*j*_) = 1 if *l*_*i*_ = *l*_*j*_, and zero otherwise. The parameter *t* is used to balance the semantic term and the color term. When the value of *t* is bigger, the superpixels are generated based on more semantic information, and more color information otherwise. According to our experience in the experiment, *t* ∈ [1,10] is appropriate to use. The improvement of superpixels is limited when the value of parameter is much smaller, and the improvement changes into decline of performance when it is rather bigger. In the experiment, we empirically choose *t* = 5.

In addition, we employ the guided filter [48] to enhance input image, to strengthen edges and obtain better superpixel.

According to above architecture, the complementation of recognition and visual attention can refine coarse segmentation, and the complementation of bottom-up manner and top-down manner can bring about further improvement. The former of the above-mentioned complementation refines semantic labels based on superpixels, which can be also regarded as a post-processing step resembling the DeepLab-CRF model. However, adding the latter, the overall architecture simulating visual mechanism is more human-like and achieves the better performance to validate our visual consideration.

More details of the baselines and GS superpixel method are provided in the following subsections.

### 2.2 Semantic labels from the DCNN-based methods

In this part, we employ the FCN [21] and DeepLab-CRF [29] methods as baselines to obtain semantic labels in a bottom-up manner.

#### FCN model

Long et al. [21] proposed to adapt contemporary DCNN into fully convolutional network (FCN) and transfer the learned representations by fine tuning towards semantic image segmentation task. The FCN can be trained end-to-end and pixel-to-pixel. Therefore, the FCN can take an input of arbitrary size and produce an output of corresponding size with efficient learning and inference.

The FCN considers AlexNet [5], GoogLeNet [7], and VGG 16-layer net [8], which are three DCNNs that performed exceptionally well in the ILSVRC image classification task. These networks initially extract feature maps by hierarchical architecture mainly including convolutional and pooling layers, and then flow into linear classifiers and output image labels. Because linear classifiers need a fixed-dimension input vector, DCNNs require fixed-sized input images and produce nonspatial outputs. The FCN discards the final classifier layers of the three DCNN classification architectures and regards these fully connected layers as a convolutional layer with kernels that cover their entire input regions. However, the convolutional parameter of DCNNs trained on the ImageNet dataset is saved. Pooling operation can also be regarded as a type of convolution. This operation converts DCNNs into FCN that can take an input image of any size and output classification feature maps that save spatial information.

**Fig 2:**
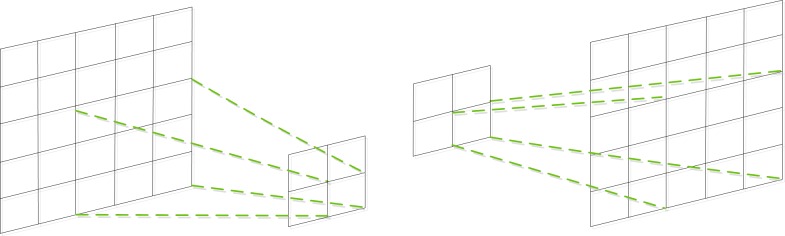
Illustration of deconvolution operations. (a) Convolution (many-to-one) (b) Deconvolution (one-to-many).

Given an input image, the FCN outputs feature maps. The size of feature maps gradually reduces in FCN because of pooling layers. The FCN appends a 1 × 1 convolution with 21 channels representing 20 classes and a background to predict the scores for each of the PASCAL VOC classes at each of the coarse output locations. Then, a bilinear upsample is employed to perform accurate prediction for the location of each pixel. For the bilinear upsample, as shown in Fig 2, the convolution layers perform the many-to-one operation. Thus, backward convolution, called deconvolution, is selected to perform the one-to-many operation with a certain convolutional kernel size and stride. Fine tuning by back propagation (BP) through entire networks is also performed on the PASCAL VOC dataset composed of the original images and supplementary images by patch sampling. Semantic segmentation results are achieved by feed-forwarding an input image in a bottom-up manner. Three DCNN classification models [5, 7, 8] are tested, and the results show that the FCN-VGG-16 model exhibits the best performance.

The FCN-VGG-16 model comprises five pooling layers with the same 2 × 2 size. Thus, the size of feature maps in the last layer is 32 times smaller than the input image and upsampling is performed 32 times to resize the feature maps to predict each pixel for the input image. As such, the FCN is called FCN-32s. However, the segmentation is global and coarse, particularly in object contour.

A skippable architecture that combines semantic information from a deep, coarse layer with appearance information from a shallow, fine layer to produce accurate and detailed segmentations is defined to further improve the semantic segmentation accuracy. In detail, deconvolution (kernel size = 4 × 4, stride = 2) is firstly performed for feature maps of conv7 layers and the output is integrated with feature maps of the fourth pooling layer. Then, upsampling of 16 times is performed to resize the integrated feature maps to predict each pixel for the input image and the FCN is called FCN-16s. On this basis, a feature map of the third pooling layers is integrated and is called FCN-8s. Experimental results indicate that the FCN exhibits excellent results with low-level features added. The output of FCN-8s illustrates an impressive performance on the PASCAL VOC benchmark and achieve 20% relative improvement to 62.2% mean IOU in 2012 test set. In our proposed architecture, we employ the best FCN-8s model based on VGG 16-layer net to perform pixel-level labeling in a bottom-up manner and introduced local detailed information in a top-down manner to refine it.

#### DeepLab-CRF model

Chen et al. [29] employed Conditional Random Field (CRF) as post-processing to improve semantic labels from fully convolutional networks, and overcame this poor localization property of deep networks by combining the responses at the final DCNN layer with a fully connected CRF [23].

Based on FCN, DeepLab-CRF similarly converts the fully connected layers of VGG-16 network into convolutional ones and runs the network in a convolutional fashion on the image at its original resolution. However, the VGG-16 has five pooling layers with the same 2 × 2 sizes, and the size of feature maps in the last layer is 32 times smaller than the input image, which is regard as the reason of coarse predicting. In order to deal with it, DeepLab-CRF develops a variation of the method and skip subsampling after the last two max-pooling layers in the VGG-16 network and modifies the convolutional filters in the layers that follow them by introducing zeros to increase their length (2× the last three convolutional layers and 4× the first fully connected layer). DeepLab-CRF employs the ‘hole algorithm’ to keep the filters intact and use an input stride of 2 or 4 pixels on feature maps instead of sparsely sampling.

To further improve coarse semantic segmentation result, DeepLab-CRF introduces the fully connected CRF. The model employs the energy function, i.e,

$$\begin{array}{l} E\left(l\right)=\sum_{i}-\log P\left(l_{i}\right)+\sum_{ij}\mu \left(l_{i},l_{j}\right)Q_{ij}\\ \;Q_{ij}=\left[\omega_{1}\exp\left(-\frac{{\left\|p_{i}-p_{j}\right\|}^{2}}{2\sigma_{\partial }^{2}}--\frac{{\left\|I_{i}-I_{j}\right\|}^{2}}{2\sigma_{\beta }^{2}}\right)+\omega_{2}\exp\left(-\frac{{\left\|p_{i}-p_{j}\right\|}^{2}}{2\sigma_{\gamma }^{2}}\right)\right] \end{array}$$ (4)

where *l* is the label assignment for pixels; *P*(*l*_*i*_) is the label assignment probability at pixel *i* as computed by DCNN; *μ*(*l*_*i*_,*l*_*j*_) = 1 if *l*_*i*_ ≠ *l*_*j*_, and zero otherwise; *Q*_*ij*_ measures the location and color difference for pairs of pixels belonging to different category. Quantitatively, DeepLab-CRF reaches 66.4% IOU accuracy in the PASCAL VOC 2012 test set compared to 62.2% IOU accuracy.

### 2.3 Local detailed information from the GS superpixel method

Felzenszwalb et al. [47] proposed an alternative graph-based approach to generate superpixels. They performed an agglomerative clustering of pixels as nodes on a graph such that each superpixel was the minimum spanning tree of the constituent pixels.

Let *G* = (*V*,*E*) be an undirected graph with vertices *v*_*i*_ ∈ *V*, the set of elements to be segmented, and edges (*v*_*i*_,*v*_*j*_) ∈ *E*, the corresponding to pairs of neighboring vertices. Each edge has a corresponding weight *w*((*v*_*i*_,*v*_*j*_)), which is a non-negative measure of the dissimilarity between neighboring elements *v*_*i*_ and *v*_*j*_. In the case of image segmentation, the elements in *V* are pixels, and the weight of an edge measures the dissimilarity between the two pixels connected by that edge. GS method employs color Euclidean distance of neighbor pixels to define weight of edge. We enhance input images by guided filter [48], and modify the measurement of edges by adding the contribution of semantic labels to construct more appropriate graph and produce better superpixels, as shown in the formula (3).

Considering the clustering strategy, they define the internal difference in a component *C* ⊆ *V* to be the largest weight in the minimum spanning tree of the component *MST*(*C*,*E*), i.e.,

$$Int\left(C\right)=\underset{e\in \left(MST,E\right)}{\max}e.$$ (5)

In addition, they define the difference between two components *C*_1_,*C*_2_ ⊆ *V* to be the minimum weight edge connecting two components, i.e.,

$$Diff\left(C_{1},C_{2}\right)=\underset{v_{i}\in C_{1},v_{j}\in C_{2},\left(v_{i},v_{j}\right)\in E}{\min}\omega \left(v_{i},v_{j}\right).$$ (6)

For small components, *Int*(*C*) is not a good estimate of the local characteristics of the data. In the extreme case, |*C*| = 1, *Int*(*C*) = 0. Therefore, they use a threshold function *τ*(*C*) based on the size of the component to adjust internal differences, i.e.,

$$\begin{array}{l} Int{\left(C\right)}^{'}=Int\left(C\right)+\tau \left(C\right)\\ \;=Int\left(C\right)+\frac{k}{\left|C\right|} \end{array},$$ (7)

where |*C*| denotes the size of *C* and *k* is a constant parameter. In practice, *k* sets a scale of observations, where a large *k* causes a preference for large components. Based on the two differences, they define the pairwise comparison predicate as follows:

$$D\left(C_{1},C_{2}\right)=\left\{ \begin{array}{l} \text{true}\;\text{if}\;Diff(C_{1},C_{2})>MInt(C_{1},C_{2})\\ \text{false}\;\text{otherwise} \end{array}\right.,$$ (8)

where the minimum internal difference, *MInt*, is defined as follows:

$$MInt(C_{1},C_{2})=\min(Int(C_{1}),Int(C_{2})).$$ (9)

The region comparison predicate if evidence for a boundary between a pair of components exists by checking if the difference between the components *Diff*(*C*_1_,*C*_2_) is large relative to the internal difference within at least one of the components *Int*(*C*_1_) and *Int*(*C*_2_).

**Fig 3:**
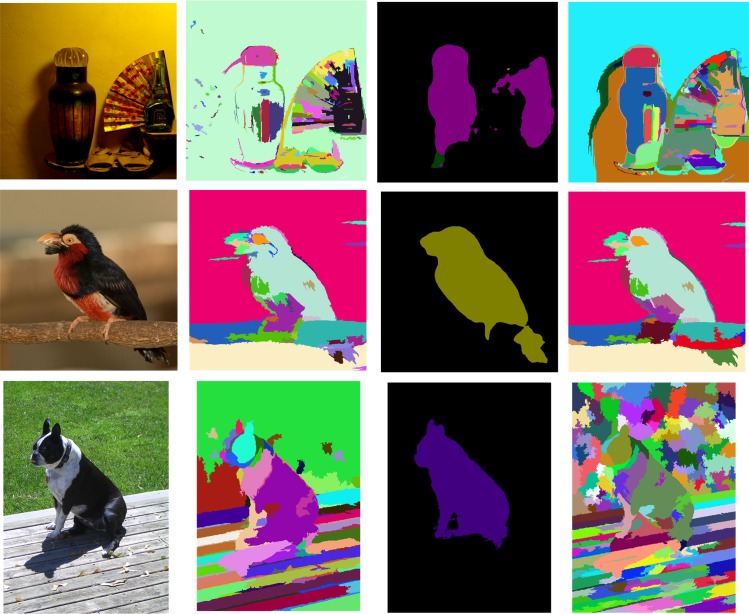
Superpixel segmentation results from the GS method. **Different colors represent different superpixels.** (a) Input image (b) Superpixels from GS method (c) Semantic labels (d) Superpixels from GS method with semantic labels.

As shown in Fig 3, the GS method adheres well to image boundaries in practice and keeps local detailed information. Based on the consideration of enhance of input image and semantic labels in the process of producing the superpixels, we obtain better superpixel segmentation.

The Complexity is *o*(*N* log *N*) and it runs fast in practice. Superpixels perform clustering of similar pixels. We could employ superpixels to refine semantic labels in a top-down manner, which is similar to the visual attention mechanism based on recognition. With the combination of bottom-up predicting and top-down refining, we improved fuzzy segmentation edge and segmentation error.

### 2.4 Method overview

Current semantic image segmentation methods based on DCNNs output coarse segmentation results in a bottom-up manner. Our architecture develops better superpixels in a top-down manner based on global semantic labels, and refines semantic labels with the introduction of local detailed information from superpixels, which is similar to human visual mechanism. We employ the FCN and DeepLab-CRF as baselines to validate our architecture.

## 3 Results and discussion

### 3.1 Results and evaluation on the PASCAL VOC 2011 and 2012 test sets

We evaluate our architecture on the PASCAL VOC 2011 and 2012 benchmark [24, 25], which respectively contains 1111 and 1456 test images. In order to compare mutual promotion of semantic labels and superpixels for segmentation results, we both test two architectures that one only uses superpixels to improve semantic labels, which is denoted by DCNN-Sp, and the other one represents overall architecture that performs mutual promotion of semantic labels and superpixels, which is denoted by DCNN-Sp-v2. In addition, we employ another superpixel segmentation method called DBSCAN [49] to sufficiently validate our proposed architecture. Similarly, DBSCAN method also generates superpixels based on the color difference between pixels and we can perform the combination according to the preceding architecture.

**Fig 4:**
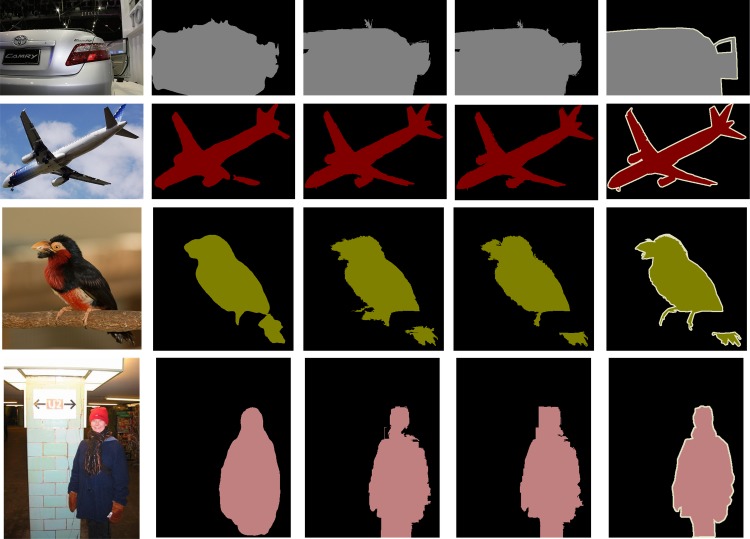
Examples that our method based on GS superpixels produced better results than the FCN model. **Different colors represent different classes.** (a) Input image (b) Segmentation results from FCN (c) Segmentation results from FCN-GS (d) FCN-GS-v2 (e) Ground truth.

**Fig 5:**
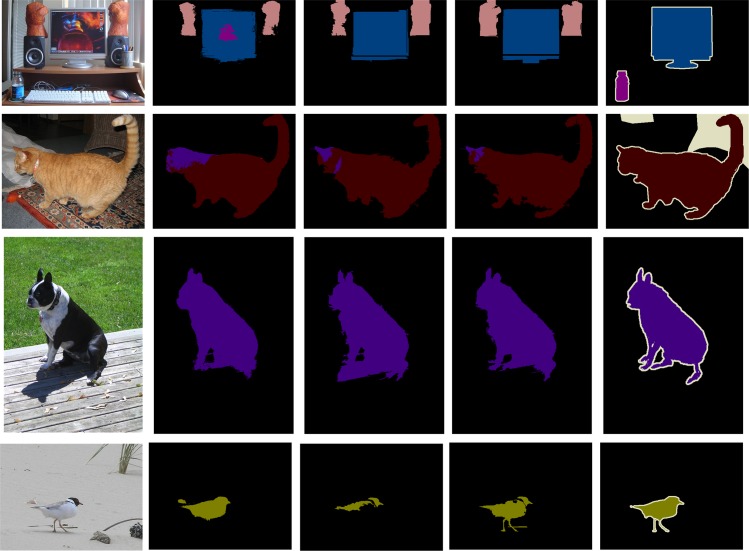
Examples that our method based on GS superpixels produced better results than the DeepLab-CRF model. **Different colors represent different classes.** (a) Input image (b) Segmentation results from DeepLab-CRF (c) Segmentation results from DeepLab-CRF-GS (d) Segmentation results from DeepLab-CRF-GS-v2 (e) Ground truth.

**Fig 6:**
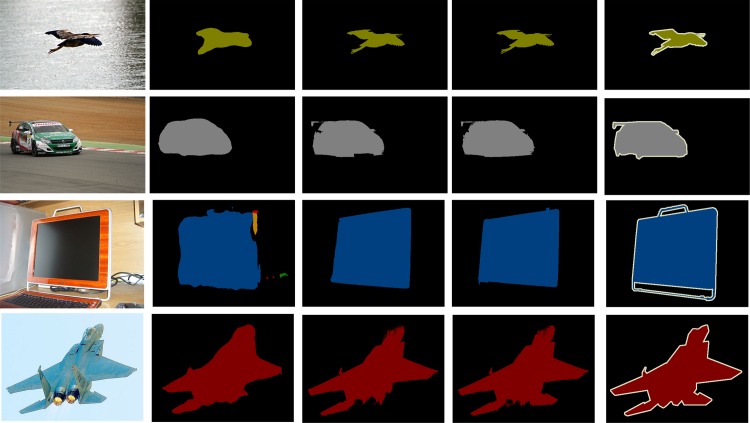
Examples that our method based on DBSCAN superpixels produced better results than the FCN model. **Different colors represent different classes.** (a) Input image (b) Segmentation results from FCN (c) Segmentation results from FCN-DBSCAN (d) FCN-DBSCAN-v2 (e) Ground truth.

In Fig 4, Fig 5 and Fig 6, we qualitatively shows that our proposed architecture improves edges, corrects error segmentation results, and achieves higher segmentation accuracy compared with the FCN [21] and DeepLab-CRF [29] on PASCAL VOC test sets.

In addition, we submitted our results to PASCAL VOC challenge performance evaluation server [25], where the IOU (intersection over union) and mean IOU are used to evaluate the performance of uploaded submissions. IOU and mean IOU are defined as follows:

$$IOU=\frac{n_{ii}}{\sum_{j}n_{ij}+\sum_{j}n_{ji}-n_{ii}}$$ (10)

$$Mean\;IOU=\sum_{i}\left(\frac{n_{ii}}{\sum_{j}n_{ij}+\sum_{j}n_{ji}-n_{ii}}\right)/n_{c}$$ (11)

where *n*_*ii*_ denotes the number of pixels of class *i* predicted to belong to class *j*, and *n*_*c*_ the number of different classes.

Table 1 quantitatively shows the performance of our proposed architecture on the PASCAL VOC 2011 and 2012 test sets, and compares it to the previous good SDS [28], and the well-known R-CNN [17]. Moreover, we obtain corresponding 2%-3% improvement based on the current state-of-art FCN-8s and DeepLab-CRF. Meanwhile, Table 2 gives detailed performance of every class on PASCAL VOC 2012 test set compared with FCN-8s and DeepLab-CRF, and it illustrates that our architecture has good generality regardless of object classes. Such results qualitatively and quantitatively validate our proposed architecture.

**Table 1:** Performance of our proposed models on the PASCAL VOC 2011 and 2012 test sets compared to other state-of-art methods.

Methods	VOC2011 test (Mean IOU %)	VOC2012 test (Mean IOU %)
R-CNN[10]	47.9	-
SDS[21]	52.6	51.6
FCN-8s[14]	62.7	62.2
FCN-GS	**64.5**	**64.4**
FCN-GS-v2	**65.1**	**65.3**
FCN-DBSCAN	**-**	**65.0**
FCN-DBSCAN-v2	**-**	**65.9**
DeepLab-CRF[22]	-	66.4
DeepLab-CRF-Sp	**-**	**67.5**
DeepLab-CRF-Sp-v2	**-**	**67.9**

**Table 2:** Evaluation results of the PASCAL VOC 2012 test set.

**Method**	**Bkg**	**Aero**	**Bike**	**Bird**	**Boat**	**Bottle**	**Bus**	**Car**	**Cat**	**Chair**	**Cow**
**FCN-8s**	91.2	76.8	34.2	68.9	49.4	60.3	75.3	74.7	77.6	21.4	62.5
**FCN-GS**	90.9	80.4	33.4	70.8	53.0	62.9	79.7	76.4	79.1	24.0	65.4
**FCN-GS-v2**	91.3	79.8	33.9	74.0	55.6	64.7	79.1	76.8	80.3	25.3	65.7
**FCN-DBSCAN**	91.2	82.2	33.1	73.3	55.6	63.3	79.8	76.1	78.3	24.5	64.8
**FCN-DBSCAN-v2**	92.0	78.5	32.4	76.9	56.6	64.6	80.6	74.7	77.8	26.7	68.4
**DeepLab-CRF**	92.1	78.4	33.1	78.2	55.6	65.3	81.3	75.5	78.6	25.3	69.2
**DeepLab-CRF-GS**	92.3	81.4	34.1	79.1	58.3	65.8	81.3	77.1	80.5	28.9	69.1
**DeepLab-CRF-GS-v2**	92.3	82.3	34.2	79.0	58.3	66.0	82.1	77.7	81.8	29.1	68.5
**Method**	**Table**	**Dog**	**Horse**	**Mbk**	**Person**	**Plant**	**Sheep**	**Sofa**	**Train**	**TV**	***Mean***
**FCN-8s**	46.8	71.8	63.9	76.5	73.9	45.2	72.4	37.4	70.9	55.1	**62.2**
**FCN-GS**	52.9	73.6	66.9	75.6	75.2	46.9	72.5	42.0	70.6	59.6	**64.4**
**FCN-GS-v2**	52.7	74.0	67.3	76.0	75.4	48.8	74.7	44.2	72.6	58.8	**65.3**
**FCN-DBSCAN**	53.6	73.9	66.6	76.9	75.0	47.6	73.2	43.4	73.2	59.0	**65.0**
**FCN-DBSCAN-v2**	52.1	73.6	68.6	78.4	76.8	54.0	76.9	46.1	72.2	57.3	**65.9**
**DeepLab-CRF**	52.7	75.2	69.0	79.1	77.6	54.7	78.3	45.1	73.3	56.2	**66.4**
**DeepLab-CRF-GS**	56.1	75.9	68.8	78.9	78.0	53.7	78.7	46.3	73.8	58.9	**67.5**
**DeepLab-CRF-GS-v2**	56.3	77.0	69.1	79.7	78.0	53.5	78.2	47.0	74.9	60.4	**67.9**

### 3.2 Discussion

As shown in Fig 4, Fig 5 and Fig 6, the DCNN-based methods obtain global recognition and identify the correct category of objects, which benefits from the powerful feature representation of DCNNs. DCNNs trained for classification in a bottom-up manner are similar to the ventral cortical area. The visual mechanism illustrates the reason why DCNN-based methods obtain success in semantic segmentation.

However, the segmentation results of the DCNN-based methods are coarse and partly present errors. From the visual mechanism, humans process dense prediction tasks by recognition in a bottom-up manner and visual attention in a top-down manner. Although having a powerful recognition capability partly similar to the ventral stream in a bottom-up manner, DCNNs lack visual attention in a top-down manner and are not enough for the semantic image segmentation task. The preceding analysis shows why DCNN-based methods lack local detailed information in a top-down manner in semantic segmentation.

In our proposed architecture, superpixels play the same part as the visual attention in human visual mechanism. For the DCNN-Sp model, superpixels are simply introduced to refine semantic labels. On one hand, the combination of DCNNs and superpixels can be also simply regarded as a post-processing step. On the other hand, DCNN-based methods obtain global recognition based on the hierarchical structure containing a large amount of parametric mapping, whereas superpixel methods achieve detailed attention by slightly non-parametric manner. So, the DCNN-Sp model can be considered as complementation of parametric and non-parametric terms. For the DCNN-Sp-v2 model, superpixels are produced in a top-down manner and it behaves more similar with the visual attention. From the comparison of DCNN-Sp and DCNN-Sp-v2, we can find that the semantic labels obtained in a bottom-up manner are useful to produce better superpixels in a top-down manner and better superpixels conversely improve the semantic labels. The more compact combination of global semantic information and local detailed information lead to more accurate segmentation results. Our architecture builds on the top semantic labels and develops accurate detailed information, then merges both, ultimately obtains better segmentation, in which, the combination of bottom-up manner and top-down manner is represented.

## 4 Conclusion

Considering the difference in classification and segmentation tasks, we find a way based on visual mechanism to explain that DCNNs in a bottom-up manner are not enough for the semantic image segmentation task, which needs not only recognition in a bottom-up manner but also visual attention in a top-down manner. We propose a kind of semantic image segmentation architecture by simulating the visual mechanism. The architecture includes the complementarity of the bottom-up and top-down manners, and complementarity of global semantic information and local detailed information. We employ coarse semantic labels from current DCNN-based methods to help produce better superpixels, and conversely utilize better superpixels to improve semantic labels. The brilliant FCN and DeepLab-CRF are used as baselines to validate our architecture. Moreover, we test and prove that the two processes are both valuable and complementary, which demonstrates that more compact combination lead to better semantic segmentation results. The experimental results qualitatively and quantitatively show that our proposed architecture improves coarse edges, corrects error segmentation, and exhibits better segmentation performance. Such a problem is expected to be solved with the improvement of DCNN performance. Better methods considering local detailed information should also highlight the architecture.

## Supporting information

S1 Dataset The file shows how to get and use the PASCAL VOC dataset employed in this paper. (DOCX)
