# Supplementary material for: A top-down manner-based DCNN architecture for semantic image segmentation
Source: PLoS One. 2017 Mar 24;12(3):e0174508. doi: 10.1371/journal.pone.0174508 (PMC5365135; doi:10.1371/journal.pone.0174508)
Supplement: S1 Dataset — (DOCX) [file pone.0174508.s001.docx]

PASCAL VOC dataset is used in this paper, and the dataset is available for open access in the official website <http://host.robots.ox.ac.uk:8080/>. In the website, you need to register an account before downloading dataset. The evaluation of new methods on these data sets can be achieved through the PASCAL VOC Evaluation Server. The evaluation server will remain active even though the challenges have now finished. In addition, you can find the guide to citation: if you make use of the dataset, please cite the following journal paper in any publications:

The PASCAL Visual Object Classes (VOC) Challenge

Everingham, M., Van Gool, L., Williams, C. K. I., Winn, J. and Zisserman, A.

International Journal of Computer Vision, 88(2), 303-338, 2010
